# Task sharing for the care of severe mental disorders in a low-income country (TaSCS): study protocol for a randomised, controlled, non-inferiority trial

**DOI:** 10.1186/s13063-016-1191-x

**Published:** 2016-02-11

**Authors:** Charlotte Hanlon, Atalay Alem, Girmay Medhin, Teshome Shibre, Dawit A. Ejigu, Hanna Negussie, Michael Dewey, Lawrence Wissow, Martin Prince, Ezra Susser, Crick Lund, Abebaw Fekadu

**Affiliations:** Addis Ababa University, College of Health Sciences, School of Medicine, Department of Psychiatry, Addis Ababa, Ethiopia; Institute of Psychiatry, Psychology and Neuroscience, Centre for Global Mental Health, King’s College, London, UK; Aklilu Lemma Institute of Pathobiology, Addis Ababa University, Addis Ababa, Ethiopia; Horizon Health Network, Dr Everett Chalmers Regional Hospital, Psychiatry, Fredericton, New Brunswick Canada; Department of Pharmacology, St Paul’s Hospital Millennium Medical College, Addis Ababa, Ethiopia; Department of Health, Behaviour and Society, Johns Hopkins School of Public Health, Baltimore, MD USA; Mailman School of Public Health, Columbia University, New York, USA; New York State Psychiatric Institute, New York, USA; Department of Psychiatry and Mental Health, Alan J. Flisher Centre for Public Mental Health, University of Cape Town, 46 Sawkins Road, Rondebosch, Cape Town South Africa; Department of Psychological Medicine, Institute of Psychiatry, King’s College London, Psychology and Neuroscience, Centre for Affective Disorders, London, UK

**Keywords:** Mental disorder, community mental health care, primary healthcare, task-shifting, sub-Saharan Africa, developing countries

## Abstract

**Background:**

Task sharing mental health care through integration into primary health care (PHC) is advocated as a means of narrowing the treatment gap for mental disorders in low-income countries. However, the effectiveness, acceptability, feasibility and sustainability of this service model for people with a severe mental disorder (SMD) have not been evaluated in a low-income country.

**Methods/Design:**

A randomised, controlled, non-inferiority trial will be carried out in a predominantly rural area of Ethiopia. A sample of 324 people with SMD (diagnoses of schizophrenia, schizoaffective disorder, bipolar disorder or major depressive disorder) with an ongoing need for mental health care will be recruited from 1) participants in a population-based cohort study and 2) people attending a psychiatric nurse-led out-patient clinic. The intervention is a task-sharing model of locally delivered mental health care for people with SMD integrated into PHC delivered over 18 months. Participants in the active control arm will receive the established and effective model of specialist mental health care delivered by psychiatric nurses at an out-patient clinic within a centrally located general hospital. The hypothesis is that people with SMD who receive mental health care integrated into PHC will have a non-inferior clinical outcome, defined as a mean symptom score on the Brief Psychiatric Rating Scale, expanded version, of no more than six points higher, compared to participants who receive the psychiatric nurse-led service, after 12 months. The primary outcome is change in symptom severity. Secondary outcomes are functional status, relapse, service use costs, service satisfaction, drop-out and medication adherence, nutritional status, physical health care, quality of care, medication side effects, stigma, adverse events and cost-effectiveness. Sustainability and cost-effectiveness will be further evaluated at 18 months. Randomisation will be stratified by health centre catchment area using random permuted blocks. The outcome assessors and investigators will be masked to allocation status.

**Discussion:**

Evidence about the effectiveness of task sharing mental health care for people with SMD in a rural, low-income African country will inform the World Health Organisation’s mental health Gap Action Programme to scale-up mental health care globally.

**Trial registration:**

NCT02308956 (ClinicalTrials.gov). Date of registration: 3 December 2014.

**Electronic supplementary material:**

The online version of this article (doi:10.1186/s13063-016-1191-x) contains supplementary material, which is available to authorized users.

## Background

### Task-sharing care to alleviate the burden of severe mental disorders in Africa

The unmet need for mental health care is high in all countries of the world but is particularly acute in low-income countries [1]. Ethiopia is typical of most low-income countries, with fewer than 10 % of people with severe mental disorders (SMD) receiving mental health care [2]. SMDs are predominantly psychotic disorders that are chronic or recurrent, have a substantial impact on affected people and their families and rank highly in terms of disease burden [3, 4]. The lack of treatment or inadequate treatment for SMDs is associated with a high level of suffering and disability [5–7], family burden [8], stigma, discrimination and human rights abuses [9, 10], out-of-pocket costs and opportunity costs to affected individuals and their families [11], poor physical health and under-nutrition [12], and premature mortality [13, 14].

Centralised services, a critical shortage of specialist mental health workers and an absence of mental health care in general health care settings are the main causes of this large treatment gap for SMDs in the low-income countries of sub-Saharan Africa [15]. In Ethiopia, outside of the capital city, Addis Ababa, mental health services are largely limited to regional cities, with most care provided within psychiatric nurse-led, hospital-based clinics [16, 17]. The majority of Ethiopia’s population, however, lives in rural areas [18]. In order to improve access to mental health care, a ‘task sharing’ approach is required. The concept of task sharing is similar to that of task shifting, defined as delegating tasks to existing or new cadres with either less training or narrowly tailored training [19, 20] but also recognises the ongoing role of specialists. For mental health care, it is advocated that primary care and general health care workers be given brief training to deliver circumscribed aspects of care for prioritised mental, neurological and substance-use disorders, with the support of specialist mental health workers who provide supervision, consultation, refresher training and referral [21].

In a series of systematic reviews conducted by the World Health Organisation (WHO), packages of mental health interventions were identified (detailed in the Mental Health Gap Project Intervention Guide: mhGAP-IG) that can be delivered in the PHC setting and have demonstrated effectiveness for a range of mental, neurological and substance use disorders [22]. The task sharing model of mental health care integrated into PHC, proposed by WHO in mhGAP, is expected to be more affordable and accessible for the majority of people with SMD in low-income countries. By locating mental health care in PHC, the potential exists for people with SMD to receive improved physical health care [23].

### The need for trials of task-sharing interventions for mental health care

Despite the potential advantages of task-sharing mental health care, little is known about how different aspects of the care needed by people with SMDs can be safely and effectively transferred to the PHC setting in a rural, low-income country setting such as Ethiopia. In a recent review of task-sharing approaches to health care in LMICs, evidence was offered that task sharing can improve the productive efficiency of services (that is, the greatest amount of care at a given quality and a given cost), although challenges regarding quality, safety and sustainability were also identified [20]. Most of the identified evaluations of task sharing were in the fields of surgery, obstetrics and HIV care, and few employed randomised, controlled trial designs. With respect to mental health care in particular, little is known about the acceptability of task sharing to service users or PHC workers [24] or the feasibility and sustainability of such a model [25, 26]. Uncertainty also exists as to whether stigma will be greater or lesser in a separate psychiatric clinic that is further from the place of residence compared to an integrated service that is in the person’s locality. The paucity of clinical trials of interventions for SMD in Africa has been highlighted, concluding that there is a pressing need for high-quality evidence from pragmatic trials with adequate follow-up periods [27]. In a consensus exercise conducted to set priorities for global mental health, the integration of mental health care into primary care was within the top five issues to be addressed to improve scale-up and impact of mental health care [28]. The TaSCS trial, therefore, addresses an important evidence gap and has the potential to inform policy initiatives to scale-up mental health care in the African region and beyond.

### Context of the TaSCS trial

The Africa Focus on Intervention Research for Mental health (AFFIRM) programme was established in 2011 with the objective of investigating strategies for narrowing the treatment gap for mental disorders in sub-Saharan Africa [29]. AFFIRM connects six countries of sub-Saharan Africa - Ethiopia, Ghana, Malawi, South Africa, Uganda and Zimbabwe - and is engaged in developing contextualised trial outcome measures [30], building capacity for intervention research, developing a collaborative network and conducting trials of task-sharing interventions in South Africa [31] and Ethiopia. Ethiopia was selected as the setting for a task-sharing trial for care of people with SMD for several reasons: 1) the Federal Ministry of Health of Ethiopia is just beginning to scale up mental health care through integration into PHC and has prioritised care for people with SMD [16]; 2) a well-described, population-based cohort of persons with standardised, clinician-defined diagnoses of SMD in Butajira, Ethiopia [2], provides a more relevant sample within which to nest a trial to inform scale-up than the usual facility-based samples; and 3) Ethiopia is a predominantly rural, low-income African country, which provides an important test case for WHO’s mhGAP.

### Rationale for a non-inferiority trial

In this trial, we propose to investigate the non-inferiority of a task-sharing model of mental health care in PHC compared to the established alternative service model within Ethiopia: a less accessible (more centralised), but more specialist, psychiatric nurse-led model of care. The psychiatric nurse-led model of care has been demonstrated to be acceptable and associated with improved clinical outcomes for people with SMD engaged in the service in this sample in Butajira, Ethiopia, thus making this an appropriate comparison model [2]. Task-sharing mental health care in PHC in Ethiopia is expected to allow more mental health care to be provided for the same cost compared to expanding specialist mental health care and is, therefore, of critical importance in addressing the high treatment gap. However, the important policy question for Ethiopia, and other low-income countries, is to establish whether or not the new task-sharing model for mental health care in PHC is good enough to meet the needs of people with SMD. At present, no evidence exists to inform this policy question. We will therefore conduct a non-inferiority trial in order to evaluate whether or not task sharing is at least no worse than specialist mental health care across a range of outcomes.

## Objectives

The overall objective of the TaSCS trial is to determine the acceptability, affordability, effectiveness and sustainability of mental health care for people with SMD, delivered by trained and supervised non-specialist PHC workers compared to an existing psychiatric nurse-led service.

The specific objectives are as follows:

1. To determine the effectiveness and cost-effectiveness of task-sharing mental health care for people with SMD with PHC, compared to psychiatric nurse-led mental health care, on the primary outcome measure of symptom severity and on a series of secondary outcome measures.
2. To examine factors influencing the implementation of the task sharing intervention and future scale-up, by examining the feasibility, sustainability, quality and safety, and by qualitative exploration of the experience of task sharing from the perspectives of service users, PHC workers and health service managers.

### Hypothesis

People with SMD who receive mental health care that is task shared with PHC will have a non-inferior clinical outcome, defined as a difference in the mean symptom score on the Brief Psychiatric Rating Scale, expanded version (BPRS-E) [32], of no more than six points higher, compared to people with SMD who receive a psychiatric nurse-led model of mental health care, after 12 months.

## Methods

### Trial design

The study is an individual level, randomised, controlled non-inferiority trial.

### Setting

The study will be conducted in the Meskan and Mareko districts of the Gurage Zone and the Silti Zone of the Southern Nations, Nationalities and People’s region, Ethiopia. The Meskan and Mareko districts have an estimated population of 159,884 and 63,436, respectively [18]. The area is predominantly rural. Most inhabitants are farmers, growing maize for subsistence and chili pepper and khat (an amphetamine-like substance) as cash crops. The main town is Butajira, located around 130 km from the capital city, Addis Ababa. Road infrastructure has expanded in the last few years, but the majority of the population do not live close to all-weather roads. The population is prone to food insecurity and was affected by famine in 1974, 1985, 1999 and 2003. Mental health research activities have been carried out in the Butajira area for the last 18 years.

Within the study site, one government general hospital is present, located in the town of Butajira. The hospital is staffed by general physicians, a surgeon and obstetrician, as well as health officers (3 to 4 years of training at BSc level) and nursing staff (2 to 3 years training at BSc and Diploma level). There are 13 health centres in the rural areas surrounding the town of Butajira. Health centres provide PHC services comprising preventive health care, treatment of acute illness and delivery services, but no in-patient care. Health centres are staffed by health officers and general nurses. Each health centre is linked to five satellite health posts, staffed by two community health extension workers and located within walking distance of most residents (most with 5 to 10 km). Health extension workers are all women from the local area who have completed high school (grade 10) and a year of training in health promotion and illness prevention. The health extension workers form an interface between the PHC system and the community, dividing their time between house-to-house visits, community awareness-raising activities and a limited range of health post-based primary care services.

### Participants

Study participants will be recruited from two sources: (1) the population-based Butajira SMD cohort study sample and (2) the Butajira psychiatric nurse-led out-patient clinic.

#### Butajira SMD cohort

The Butajira SMD population-based cohort was established between 1998 and 2001 [5]. People with possible SMD were identified in two ways: (1) through a house-to-house survey covering 68,378 people using the Composite International Diagnostic Interview (CIDI) [33] as a screening tool, and (2) through community key informants, who had been trained using vignettes describing typical presentations of SMD [34]. All potential cases were invited for a second phase clinician assessment conducted by an Ethiopian physician using the Schedule for Clinical Assessment in Neuropsychiatry (SCAN) [35]. Of the 2285 SCAN assessments conducted, 844 people were diagnosed as having SMD and recruited into the cohort. Incident cases (n = 75) from the geographical catchment area over the next 2 years also underwent confirmatory diagnosis with SCAN and were recruited into the cohort, giving a total of 919 people with SMD: 359 with schizophrenia or schizoaffective disorder, 345 with bipolar disorder and 215 with severe major depressive disorder [13]. After 10 years (2011/2012) of follow-up, a clinician assessment was carried out using the Longitudinal Interval Follow-up Evaluation (LIFE) chart [36]. At that time, loss to follow-up from the cohort was as follows: 121 had died, 112 had refused to continue study assessments, 15 were vagrant and 70 had changed address and were not contactable. Study participants were more likely to be lost to follow-up if they were male, unmarried, had a diagnosis of schizophrenia and were more severely unwell at baseline, although the differences were small. However, loss to follow-up was not associated with age, literacy or employment status. The Butajira SMD cohort is one of the very few population-based studies of SMD from a LMIC. Even with the potential attrition bias, the Butajira SMD cohort offers a unique opportunity to obtain a sample that is free from the substantial selection bias associated with facility-based recruitment and is potentially more generalisable to people with SMD, who would access task-shared mental health care in PHC.

#### Butajira hospital psychiatric out-patient clinic

In Butajira, the general hospital has a psychiatric out-patient clinic run by psychiatric nurses. At present this clinic is the only mental health care available within the districts covered by the TaSCS trial, as is the case in most of Ethiopia [37]. Since completion of the Butajira SMD cohort recruitment, additional people with SMD living in the Butajira SMD/TaSCS recruitment area have sought care from this clinic because they are (1) incident cases, (2) missed cases from the original recruitment, or (3) they have migrated into the area. If the TaSCS trial is unable to recruit a sufficient sample size from the Butajira SMD cohort, consecutive attendees at the Butajira hospital psychiatric clinic will be screened for eligibility.

Recruitment into the trial will be divided into two phases. In Phase 1, only people with SMD who are clinically stable will be recruited. Three months after beginning recruitment, the Data Safety and Monitoring Board (DSMB) will review adverse events, including non-engagement with the service, disengagement from care and evidence of potentially dangerous prescribing, before Phase 2 recruitment is permitted. In Phase 2, people with SMD who have more complex needs or who are clinically unstable will be recruited.

#### Eligibility criteria for both phases of recruitment

To be eligible, participants must meet the following criteria:

1. A Diagnostic and Statistical Manual of Disorders (version IV) [38] diagnosis of schizophrenia, schizoaffective disorder, bipolar disorder or major depressive disorder made using a standardised, semi-structured clinician-administered assessment.
2. Ongoing need for mental health care, defined as active prescription of psychotropic medication or prescription of psychotropic medication within the preceding 2 years, or the person being in partial or full relapse.... These severity criteria were developed to reflect realities of service provision in Ethiopia: a person with SMD who has been well off medication for at least 2 years is unlikely to engage actively with treatment services or be considered a priority by PHC workers [39].
3. Not treated with a mood-stabiliser, second generation antipsychotic medication or thioridazine (still used in clinical practice in Ethiopia), as these medications are not available routinely in the study area.
4. Living in the catchment area of one of the health centres in the trial study site, excluding Butajira health centre catchment area. Butajira health centre is excluded because formative work indicated a lower acceptability of randomization in those who live in close proximity to the psychiatric nurse-led unit in Butajira hospital [40].
5. Planning to continue living in the area for the duration of the trial (at least 18 months).
6. The age criterion for recruitment into the original Butajira SMD cohort was 18 years or older, but participants are now older than this in view of the length of time that the cohort has been running. Therefore, participants recruited from the Butajira hospital psychiatric clinic will be required to be 25 years or above in order to ensure comparability.
7. Participants recruited from the Butajira hospital psychiatric clinic will also be required to have been in contact with mental health services for at least 2 years to ensure comparability with the Butajira SMD cohort recruits.
8. Competent in the Amharic language. There is high ethnic and linguistic diversity within the study site, but the majority of people speak Amharic, the official language of Ethiopia, even if it is not their first language.
9. Not expressing active suicidal intent.
10. Not receiving treatment for a co-morbid medical condition at Butajira hospital.
11. Not pregnant while receiving depot medication.
12. Able to give informed consent or, if lacking capacity to consent and no evidence of refusal, guardian permission obtained.

#### Additional eligibility criteria for Phase 1

1. Not pregnant or breast-feeding.
2. No co-morbid complex or unstable medical condition interfering with management of SMD.
3. Not fulfilling criteria for a diagnosis of alcohol or khat use disorder in the past 12 months.
4. Not prescribed depot antipsychotic medication at the time of assessment.
5. Stable clinical condition: either in remission from SMD or with residual symptoms that have been stable over the preceding three months.
6. No suicide attempt in the past three months.
7. Not restrained.

The eligibility criteria for the trial are designed to ensure that the findings can be generalised to people with SMD in Ethiopia who will be receiving mental health care integrated into PHC as part of the planned scale up of mental health care by the Federal Ministry of Health. The population will also be comparable to the Ethiopian studies that have shown the psychiatric nurse-led care (control arm) to be clinically effective [2, 41]. The operationalisation of eligibility criteria and their assessment is detailed in Additional file 1.

### Recruitment and screening

See Fig. 1.

**Fig. 1:**
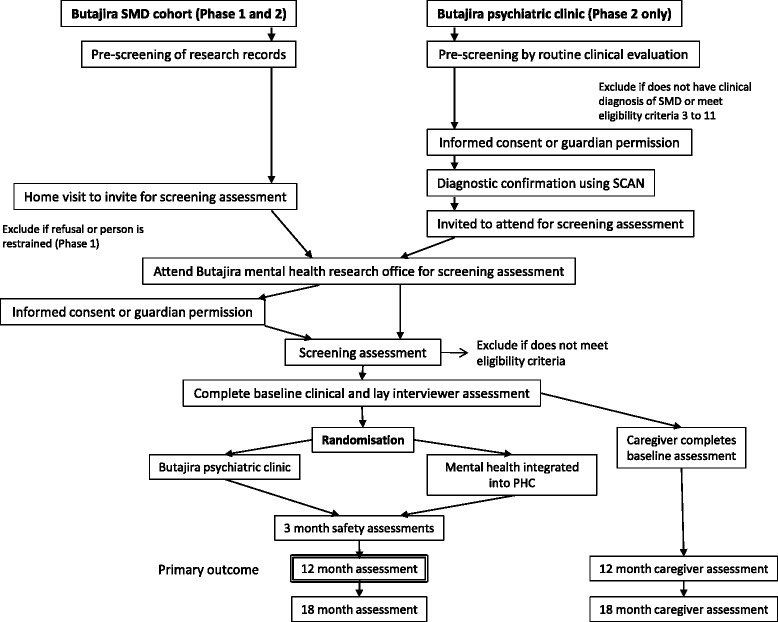
Flow chart of the Task Sharing for the Care of Severe Mental Disorders in a Low-income Country (TaSCS) trial

#### Phase 1 (Butajira SMD cohort only)

Because the last formal assessment of all cohort participants was in 2011/2012, a process of pre-screening will take place prior to starting recruitment into the trial. A pre-screen will be completed from the project clinical records and in consultation with project outreach workers. At this stage, people who are known to be prescribed psychotropic medications that are unavailable outside of Addis Ababa or who are taking depot medication, those who are documented to be pregnant or breast-feeding and those who are documented to have relapsed (within the previous three months) will be excluded. These criteria provide the sampling frame for Phase 1.

Each person with SMD included in the sampling frame for Phase 1 will be ordered randomly using computerized generation of random numbers. This random order will form the sequence in which potentially eligible patients will be invited for assessment by project outreach workers who are well known to the patients and their families. At the time of invitation, the project outreach workers will also identify the appropriate caregiver to accompany the patient to the baseline assessment. The recruitment and assessment will take place at the mental health research office in Butajira where there will be full access to the clinical notes. The target sample size for Phase 1 is 124.

For people with SMD who attend a trial assessment, a psychiatric nurse will conduct an assessment of capacity to consent to participate in the trial. After obtaining informed consent from the patient (if they have capacity) or permission from the guardian (if they lack capacity and are not refusing to participate), the initial baseline assessment measures will be administered by psychiatric nurses. On the basis of this initial assessment, eligibility for inclusion within Phase 1 of the trial will be determined. All patients who are eligible will then complete further assessments administered by non-clinical data collectors. The caregiver of the patient will also be invited to participate in the study, their consent obtained and measures administered by non-clinical data collectors. Once both patient and caregiver assessments are complete, randomization will take place.

#### Phase 2 (Butajira SMD cohort and Butajira hospital psychiatric clinic)

Phase 2 recruitment will be from the sampling frame of the Butajira SMD cohort in the first instance, only progressing to the Butajira hospital psychiatric clinic if the required sample size has not been reached.

1. Butajira SMD cohort

Phase 2 recruitment will proceed as for Phase 1. Pre-screening will only exclude women who are recorded as being pregnant at the same time as being prescribed depot and people who have already been recruited into the trial. The target sample size for Phase 2 is 200.

(2) Butajira hospital psychiatric out-patient clinic

If it is not possible to obtain the sample size for Phase 2 from the Butajira SMD cohort alone, we will expand recruitment to the Butajira hospital out-patient clinic. Unlike participants recruited from the Butajira SMD cohort, these recruits will not have undergone diagnostic research assessments. This adds an additional step to the recruitment process. Consecutive attendees who have a clinical diagnosis of SMD (as defined previously) will be pre-screened by the Butajira hospital psychiatric nurses. If the person fulfils all of the pre-screening criteria, they and their accompanying caregiver will be invited to meet with the trial psychiatric nurse who will evaluate the capacity of the person to consent to participate in the trial. If the person has capacity and gives informed consent or the eligible caregiver gives permission, they will be undergo a standardised, semi-structured diagnostic evaluation by a mental health professional [35, 42]. If the person fulfils criteria for DSM-IV diagnoses of schizophrenia, schizoaffective disorder, bipolar disorder or depressive disorder, they will be invited to return to the Butajira mental health research office on a convenient date when they can undergo the full screening and baseline assessment accompanied by an eligible caregiver.

### Randomisation and service allocation

#### Sequence generation and allocation concealment

A computerized system will be used to generate a randomization list. The randomization will be stratified by health centre catchment area. Within each health centre catchment area, randomization will be organized with permuted blocks of random size so that an equal number will be randomized to the new intervention arm and to the active control arm. From the randomization list, labelled and sealed envelopes will be prepared containing cards with intervention allocation. The sealed envelopes will be handed over to independent clinicians working in Butajira hospital and will be kept in a secure place.

#### Intervention allocation

Upon completion of the baseline assessment, participants will be accompanied to Butajira hospital. In a fully private location, the independent clinician will open the next sequential envelope for the health centre catchment area where the person resides and inform the participant of the service to which they have been allocated (that is, psychiatric nurse-led care or care from the health centre staff).

### Masking

Outcome assessors and trial investigators will be masked to the intervention allocation of participants, but it is not possible for trial participants and clinicians involved in delivering the intervention service to be masked. To minimise the possibility of unmasking of outcome assessors, 1) outcome assessments will be carried out by project-employed mental health professionals and lay interviewers in a location that is independent of and geographically separated from the health service; 2) the outcome assessors will not work in the trial districts; 3) when patients and caregivers are registered for their follow-up assessments, they will be instructed not to tell the interviewer where they are receiving mental health care; 4) selected trial clinical assessments will be observed by non-project psychiatrists to ensure that ratings are being conducted without bias; and 5) at the end of each follow-up assessment, the assessor will be asked whether they have become unmasked to patient allocation group during the course of the assessment. This information will be used to conduct a sensitivity analysis to investigate any evidence of bias. Data analysis for the primary outcome will be carried out by an independent organization, and those involved in data analysis will be masked to the intervention status of the participant.

### Interventions

#### Intervention arm

The intervention is a task sharing model of mental health care for people with SMD integrated into PHC, as recommended by the WHO in the mental health Gap Action Programme (mhGAP) [21] and endorsed by the Ethiopian Federal Ministry of Health [16]. Adaptation of the task-sharing model for the study setting was informed by extensive formative work and community consultation in the study site [40] and from findings arising from a sister project implementing integrated mental health care in a neighbouring district [43, 44].

In the TaSCS intervention, PHC-based health centre nurses and health officers will be trained to deliver the mhGAP packages of mental health care, supported by community-based health extension workers. The training and supervision requirements are summarised in Table 1. As all trial participants have an existing care plan used by project psychiatric unit staff, this will be transferred to PHC when a patient has been randomised to a PHC site. A minimum of monthly, facility-based follow-ups will be stipulated during the initial phase, and thereafter, 3-monthly follow-ups, although contacts can be more frequent depending on patient need. The current outreach by project workers will be replaced by outreach by health extension workers, who are required to make house-to-house visits every 3 months, as part of their health promotion and illness prevention responsibilities. Psychiatric nurse-led mental health support will be provided to the PHC health officers and nurses through (1) supervision including case reviews, discussion of difficult cases, developing supportive relationships with the primary health care provider and trouble-shooting; (2) emergency consultation with project psychiatric nurses; and (3) referral for a second opinion or for a period of follow-up by the specialist team in Butajira psychiatric unit, as per mhGAP guidelines. Psychiatric nurses can in turn consult project psychiatrists. The roles of the PHC workers in the task shared model are summarised in Additional file 2.

**Table 1:** Task sharing intervention for the Task Sharing for the Care of Severe Mental Disorders in a Low-income Country (TaSCS) trial

Recipients	Intervention	
	Training	Ongoing support and structures
Health centre nurses and health officers	9 days of training in mhGAP-IG packages (4 days of base course + 5 days of on-the-job training)	Phase I: Support and supervision by project psychiatric nurse tapering down from weekly to bi-weekly
	Delivered by project psychiatric nurse supported by project psychiatrist	Phase 2 onwards: Monthly support and supervision by project psychiatric nurse
	Pre-study run-in phase: on-the-job training with patients with SMD who volunteer for treatment in PHC, delivered by project psychiatric nurse	Supervision sessions will include discussion of all cases presenting with suicidal ideation or a psychiatric emergency, discussion of complex cases, developing supportive relationships with the general health care provider and trouble-shooting.
	1-day refresher training at 6 and 12 months	Emergency consultation with project psychiatric nurse
		Referral for specialist review at Butajira psychiatric unit
		Register of appointment times for persons with Severe Mental Disorder needing ongoing care
Health extension workers	2-day training course based on Ministry of Health training materials^*^.	Monthly supervision by health centre-based supervisor
	Delivered by project psychiatrist	Consultation with health centre supervisors
District health office and community stakeholders	Stakeholder workshop facilitated by project psychiatrists	Regular meetings of the trial advisory board and one-to-one liaison of project psychiatric nurse with district health office heads

* http://www.open.edu/openlearnworks/course/view.php?id=19%3f

#### Active comparison arm

The active comparison sample for the TaSCS trial is a psychiatric nurse-led, centralised model of out-patient specialist mental health care augmented by community outreach by lay workers as part of the Butajira SMD study. There are 57 psychiatric nurse-led units across Ethiopia providing the lion’s share of specialist mental health care in the country [37]. Psychiatric nurse-led clinics constitute the only model of mental health care available in rural areas in Ethiopia. The comparison arm of the TaSCS trial can be considered to be an enhanced version of specialist mental health care, in that community outreach is provided. Evaluation of the longitudinal illness course of patients in the Butajira SMD study showed improved outcomes for those who engaged with the service compared to those who did not engage [2].

The Butajira hospital psychiatric nurses are able to prescribe psychotropic medication (including depot antipsychotic medication), monitor and intervene against side effects, diagnose co-morbid psychiatric conditions and provide simple psychosocial interventions. Follow up will be arranged on the basis of need, but at least a face-to-face assessment every three months. Consultation with project psychiatrists or referral for in-patient psychiatric care (in Addis Ababa) will be as indicated.

The psychiatric nurses will receive 2 days of refresher training in order to familiarise them with the WHO’s evidence-based guidelines for management of mental disorders (mhGAP-IG). They will also receive refresher training on the management of SMD in pregnant and breastfeeding women.

### Fidelity

The competence of the PHC workers to deliver mental health care will be evaluated through pre- and post-training assessments of knowledge and attitudes [45] towards mental health, post-training evaluation of clinical skills [46] and structured supervision reports from the project psychiatric nurses. The fidelity of the task shared mental health care to the mhGAP intervention packages will be measured through structured evaluation of clinical follow-up forms by independent psychiatrists, including prescribing, risk assessment, psychoeducation and symptom review. Medication is being supplied by the trial, but a record will be kept of any interruptions to supply.

The fidelity of the active comparison specialist mental health care model to evidence-based mental health care will be measured through structured evaluation of clinical follow-up forms by independent psychiatrists, as for the intervention arm.

### Outcomes

The trial outcomes are summarised in Table 2.

**Table 2:** Summary of outcomes measures for the Task Sharing for the Care of Severe Mental Disorders in a Low-income Country (TaSCS) trial

	Outcome	Measure	Timing
Primary outcome			
1	SMD symptom severity	BPRS-E	Baseline, 12* and 18 months
Secondary outcomes			
1.	Functional impairment^±^	WHODAS 2.0 (12-item)	Baseline, 12 and 18 months
		Local functioning scale	Baseline, 12 and 18 months
2.	Relapse of mental disorder	LCS	Baseline, 12 and 18 months
3.	Service use costs	CSRI	Baseline, 12 and 18 months
4.	Satisfaction with mental health care	Mental health service satisfaction scale (MHSSS)	Baseline, 12 and 18 months
		Qualitative in-depth interviews	From 6 months post-randomisation
5.	Nutritional status	Body Mass Index	Baseline, 12 and 18 months
6.	Service use for physical health conditions	CSRI	Baseline, 12 and 18 months
7.	Medication side effects	ASC	Baseline, 12 and 18 months
8	Patient engagement and adherence	Medication Adherence Measure Clinic attendance	Baseline, 12 and 18 months
9.	Perceived stigma	ISMI	Baseline, 12 and 18 months
10.	Restraint^±^	Proportion chained, restrained or confined in last month	Baseline, 12 and 18 months
11.	Quality of care	Document review	Monthly for 3 months, 6, 12 and 18 months
13.	Adverse events (AEs)	LCS and project reporting mechanisms for AEs	Continuous for serious AEs 6-monthly for other AEs
Mediating variable			
1.	Therapeutic alliance	HAQ	Baseline, 12 and 18 months
Caregiver outcomes			
1.	Perceived stigma	FIS	Baseline, 12 and 18 months
2.	Caregiver burden	FIS	Baseline, 12 and 18 months
3.	Time burden of caring and opportunity costs	CSRI	Baseline, 12 and 18 months
Potential confounding variables			
1.	Sociodemographic characteristics	Structured self-report of age, sex and marital status	Baseline
2.	Socio-economic status	Structured self-report of educational level, occupational status, food insecurity and family size	Baseline
3.	Alcohol use disorder	FAST	Baseline
4.	Khat use disorder	CIDI substance use module	Baseline
5.	Medical co-morbidity or physical disability	Structured self-report	Baseline

* Primary outcome; ^±^ proxy version also administered to caregivers. BPRS-E, Brief Psychiatric Rating Scale, expanded version [6, 60]; WHODAS 2.0, World Health Organisation Disability Assessment Schedule, Version 2.0 [61]; LCS, Life Chart Schedule [62]; CSRI, Client Service Receipt Inventory [63, 64]; ASC, Antipsychotic Side Effect Checklist [65], Medication Adherence Measure [66]; ISMI, Internalised Stigma of Mental Illness [67]; HAQ, Helping Alliance Questionnaire [68]; FIS, Family Interview Schedule [69]; FAST, Fast Alcohol Screening Test [70]; CIDI, Composite International Diagnostic Interview [33]

#### Primary outcome

The primary outcome is non-inferior symptom level, defined as no more than a six-point higher mean score on the BPRS-E [47]. The BPRS-E is a 24-item, clinician-rated scale that was originally developed for detection of symptom change in persons with persistent SMD [47]. Each item is rated on a scale from 1 to 7 (1 = not present; 2 = very mild; 3 = mild; 4 = moderate; 5 = moderately severe; 6 = severe and 7 = extremely severe). The maximum total score is 168. The BRPS-E focuses on symptoms of psychosis but also has items covering the symptom domains of somatic concerns, anxiety, depression and mania. The total BPRS-E score is sensitive to change in treated in-patient populations of persons with persistent SMD, including those with diagnoses of schizophrenia, bipolar disorder and major depressive disorder [48]. The BPRS-E has also been used in treatment trials with out-patient populations of persons with SMD [49, 50] and has been used in Ethiopia previously [51]. The factor structure and sensitivity to change of the BPRS-E has been shown not to vary significantly across diagnostic groups [48]. Inter-rater and test-retest reliability, as well as internal consistency, have been shown to be high [52].

The non-inferiority margin for the primary outcome needs to be defined in relation the minimal clinically important difference (MCID) [53]. Previous studies have defined clinically significant change in score on the BPRS-E in different ways. Using a statistical approach, the ‘reliable change index’ [54] for change in BPRS-E score from beginning to end of an in-patient admission in persons with SMD equated to 18 points [55]. However, change in symptoms in a person with acute psychosis receiving in-patient care is likely to be larger than the MCID and not applicable to the out-patient sample of people with SMD to be included within this sample. In a study looking at change in symptoms in response to a psychological intervention for an out-patient sample of people with chronically symptomatic schizophrenia, a mean change of 7.9 points on the BPRS-E was considered clinically important [49]. A mean change of 7.9 points equates to a standardised effect size of around 0.50, which we take to be the MCID. In line with recommendations for non-inferiority trials, the non-inferiority margin for this trial will be 75 % of the presumed MCID, that is, a six-point difference in mean BPRS-E scores between arms of the trial [53, 56]. A six-point difference on the BPRS-E equates to a standardised effect size of 0.4.

#### Secondary outcomes

See Table 2.

A wide range of secondary outcomes are being assessed in view of the importance of exploring the full range of possible harms when conducting a non-inferiority trial. The secondary outcomes are functional impairment, relapse of SMD, service use costs, satisfaction with mental health care, nutritional status, service use for physical health conditions, disengagement from care, non-adherence with medication, experience of side effects, perceived stigma, experience of being restrained, quality of care and adverse events. Caregiver outcomes are perceived stigma, family burden, time burden and opportunity costs of care giving.

#### Potential confounders

In order to be able to adjust for potential confounding in case of randomly unequal distribution of variables, baseline measurement of the following variables will be carried out: socio-demographic characteristics, socio-economic status, alcohol use disorder, khat use disorder and medical co-morbidity and physical disability. See Table 2.

### Sample size calculation

The following formula was used to calculate the required sample size (n):

$$ n={2}^{*}{\left[\sigma /\Delta \right]}^2\;{\left({Z}_{\alpha }+{Z}_{\beta}\right)}^2, $$

where delta = non-inferiority margin (mean BPRS-E scores of six points), sigma is the standard deviation of the BPRS-E score (estimated standard deviation of 15 [57]), Z_alpha_ is the standardised mean difference for the probability of type 1 error (alpha = 0.05) and Z_beta_ is the standardised mean difference for the probability of type 2 error (beta =0.1 (that is, power of 0.9). Therefore, a sample size of 107 persons per arm of the trial (that is, a total of 214) is required [58]. No published studies from Ethiopia are available to guide us in our estimation of the intra-cluster correlation (ICC) for study subjects receiving treatment from a specific health centre. We therefore estimate the ICC to be 0.01, which is the median ICC from a review of 31 cluster-based interventions in PHC [59]. Given that there are 13 health centres within the three districts covered by the Ethiopia TaSCS trial, the design effect will be 1 + (n-1)*ICC (where n = number of subjects per cluster). Therefore, the design effect = 1 + (17–1)*0.01 = 1.16. Assuming a worst-case scenario of 30 % loss to follow-up, the required sample size = 214 * 1.30 * 1.16 = 323 participants. For equal numbers in each arm of the trial, the total sample size will be 324 (n = 162 in each arm).

### Data management and analysis

#### Data management

A data management plan was developed to specify all procedures relating to the handling of trial data. Management of data for the masked outcome data will be handled by the Clinical Trials Unit at the Armauer Hanssen Research Institute (AHRI) with support from the trial statisticians. Data will be doubled entered from the Patient Report Forms into an electronic Case Report Form. Trial data with unmasking potential (process data and data pertaining to adverse events) will be handled by the Butajira mental health research office following the same standardised operating procedures as for the Clinical Trials Unit. Data cleaning based on frequency distributions and logic checks will follow standard procedures with reference to source documents as required.

#### Data analysis approach

Data analysis will follow a detailed statistical analysis plan that specifies all planned analyses. Data analysis will take place using Stata Version 13 (Stata Corporation, College Station, Texas, USA) under the direction of the trial statisticians. All analyses will be masked to study arm until the analysis is finalized and approved by all investigators. Descriptive analyses will include frequency distributions and measures of central tendency and dispersion, as appropriate, with 95 % confidence intervals. Bivariable comparisons will employ χ^2^, Fisher’s exact, Student’s t- or rank-sum tests, as appropriate. All statistical tests will be one-sided at α = 0.05, as we are testing a hypothesis of non-inferiority and not superiority or equivalence.

Analysis will be by intention-to-treat, that is, on the basis of the group to which the participant is allocated randomly at the beginning of the trial. Intention-to-treat has the potential to lead to false acceptance of the hypothesis of non-inferiority in the presence of a true difference if there is a large cross-over from the primary care to the psychiatric nurse service [60]. Therefore, the intention-to-treat analysis will be presented together with the percentage of participants who deviate from the protocol in the following ways: (1) cross-over protocol deviation which is indicated by the percentage who attend for more than two consecutive visits at the service they were not randomised to receive, unless they were referred to that service, and (2) disengagement from care protocol deviation, which is the percentage who miss more than 50 % of their scheduled health facility appointments (not attending within 2 weeks of the appointment date). Per protocol analyses will also be conducted, and the findings presented as a sensitivity analysis. Account will be taken of clustering at the level of the health facility.

Analysis will be carried out on the complete data set at 12 and 18 months. For the primary outcome time-point of 12 months, it would not be appropriate to carry forward the last observation to replace missing data as this would have taken place at the baseline of the study and could, therefore, increase the chance of non-inferiority even in the presence of a difference between the allocated services.

If more than 15 % of the primary outcome scores are missing, we will conduct multiple imputation analysis (including age, sex, baseline symptom severity and substance use disorder) in the model and present this as an exploratory analysis alongside the analysis on the complete dataset.

#### Interim analysis

An interim analysis will be conducted at 12 months. The DSMB will assess the primary outcome (BPRS-E score) and adverse events by arm of the trial and decide if there is evidence of inferiority of one arm, guided by the definition of clinical inferiority used for the primary hypothesis.

#### Analysis of primary outcome

The analysis for the primary outcome will be carried out by AHRI in order to ensure masking of the treatment group. The primary outcome measure (change in BPRS-E score) will be analysed using linear regression adjusting for baseline symptom severity. Statistical tests will be one-sided, reflecting our interest in testing the hypothesis that task-shared care is non-inferior. The validity of regression assumptions will be checked using residual plots. For the purpose of safety monitoring, the Phase 1 primary outcome at 12 months will be analysed as an interim analysis. For the purpose of hypothesis-testing for the main trial, Phase 1 and Phase 2 primary outcomes at 12 months will be combined. Both crude and adjusted (for baseline symptom severity, age and sex) effect sizes will be presented for the primary analysis. Exploratory analysis of the non-inferiority of the new intervention in the Phase 1 and Phase 2 participants will be carried out separately.

#### Analysis of secondary outcomes

These analyses will be carried out by the trial statisticians. The planned approach for data analysis of secondary outcomes is outlined in Additional file 3. Multivariable analysis will be used to adjust for the following potential confounding variables measured at baseline: age, sex, socioeconomic status, substance use and symptom severity.

#### Economic analysis

The economic analysis will measure both the costs associated with a task-sharing model of care, as well as the consequential impact on service use. Local unit costs will be applied to resources used so as to estimate changes in services costs in both intervention and comparator groups. Time lost from work by people with SMD and their caregivers will be valued using appropriate wage rates, adjusted to take account of patterns of employment in an agrarian economy. Costs will be analysed using multiple regression analysis so that the effect of the intervention on costs can be estimated whilst adjusting for differences in participant characteristics. Non-parametric bootstrap methods will be used if the residuals of the regression model are non-normally distributed. Further regression models will include the main clinical measures so that the relationship between costs and outcomes can be assessed. A cost-effectiveness analysis will be carried out. Differences in cost and primary outcome measure (BPRS-E) that take into account the uncertainty around point estimates will be plotted and subsequently presented as a cost-effectiveness acceptability curve.

### Ethical considerations

Ethical approval for the trial has been obtained from the Institutional Review Board of the College of Health Sciences, Addis Ababa University (Reference Number 030/12/Psy), the AHRI-ALERT Ethics Review Committee (Reference Number P037/13), the National Research Ethics Review Committee of Ethiopia (Reference Number 3.10/758/07), the Food, Medicine and Health Care Administration and Control Authority (Reference Number 02/6/22/13), the University of Cape Town Human Research Ethics Committee (Reference Number 226/2011) and the National Institute of Mental Health Data Safety and Monitoring Board (DSMB).

The trial will include people with SMD who lack capacity to consent to participate in the trial as long as they are not actively refusing and they have a guardian who gives permission. Given the lack of knowledge on task-sharing care for people with SMD, and the intention of the Ethiopian Ministry of Health to implement this new model of service provision, it is crucial that people with SMD who are representative of those who will receive the task sharing model in the future have the opportunity to participate in the study. In participants who lack capacity at baseline, the capacity to consent will be reviewed at follow-up assessment time-points. If the participant regains capacity, they will be informed about the trial and will only continue in the trial if they give informed consent. No out-patient treatment from non-specialist mental health workers will be provided to people with any mental disorder against their will, except for management of acute behavioural disturbance where the person poses an imminent risk to themselves or others.

There is genuine uncertainty as to whether or not a task-sharing model of mental health care for SMD can be as effective as mental health care delivered by mental health specialists, but also no evidence that it is any worse, supporting a position of clinical equipoise. In the absence of any difference in effectiveness of care, a task-sharing model of care may be preferred through being locally available, even if the practitioners delivering the care are not specialists.

During the trial period participants will directly benefit from free treatment for both mental and physical health problems. Participants will be compensated for transport and their time when attending for trial-related assessments. Transport costs for attending Butajira hospital will also be covered.

We anticipate that the main risks for those allocated to the task-sharing model of mental health care in PHC will be modest: 1) moving from a familiar arrangement to a new treatment setting may be unsettling at the beginning, and therefore, monthly reviews will be scheduled at the beginning of the trial in order to facilitate engagement; and 2) there is a risk of receiving inferior care, which will be detected through monitoring of clinical records, supervision and the 12 month interim analysis. It is not anticipated that either arm of the trial will be associated with an increase in serious or other adverse events; however, procedures are in place for minimising the risk of adverse events and ensuring monitoring, reporting and management of adverse events to the DSMB and other regulatory bodies. The risk of suicide will be minimised by excluding people who express active suicidal intent at the time of recruitment and requiring clinicians to use structured clinical follow-up forms that prompt for screening for suicide risk. The phased nature of recruitment is also designed to reduce risk of adverse events by ensuring that PHC workers have gained competence in managing care of people with stable or uncomplicated SMD before they take on the care of people with more complex illness. The occurrence of serious or other adverse events will be formally reviewed at 12 months as part of the interim analysis.

All trial-related assessments will take place in a location that respects the participant’s privacy. The confidentiality of the participants will be respected, and the names of the patients will not be quoted or published.

The trial will be subjected to rigorous on-site monitoring of safety and quality at study initiation and thereafter twice per year by an independent external monitor reporting to the NIMH DSMB, in addition to routine reporting to the NIMH DSMB twice per year.

## Discussion

A pressing need exists to scale up evidence-based packages of mental health care in LMICs and thereby improve the clinical, functional and social outcomes of people with mental disorders. Alongside this imperative runs a critical need to evaluate the success of the task-sharing models of mental health care, which are proposed as solutions to the treatment gap for mental disorders. Human resource and health system constraints mean that interventions that are demonstrated to be efficacious when evaluated individually cannot be assumed to be effective when delivered as part of a service in more real-world conditions. The dearth of high-quality mental health service trials from LMICs generally, and from low-income countries in particular, is a serious impediment to the successful scale-up of mental health care. The TaSCS trial seeks to provide high-quality evidence that will be timely for informing mental health care scale-up in other rural, low-income countries, as well as contributing to the international discourse on task sharing as an acceptable model of care for people with SMD.

### Trial status

Recruitment started on 13 March 2015 and is ongoing.

## Additional files

Additional file 1:
**Operationalisation and assessment of eligibility criteria.** Description: Specifies the eligibility criteria, together with the measures for assessing eligibility. (DOCX 14 kb) Additional file 2:
**Roles of primary care workers in the task sharing intervention service.** Description: Describes the roles that the primary care workers are being trained to do within the new task-shared service. (DOCX 12 kb) Additional file 3:
**Planned data analyses for secondary outcomes.** Description: Gives details of the analysis plan for secondary trial outcomes. (DOCX 12 kb) Additional file 4:
**CONSORT Statement 2006 - Checklist for Non-inferiority and Equivalence Trials.** Description: Gives detail about where the required CONSORT information is located within the manuscript. (DOC 67 kb)

## Abbreviations

mhGAP: mental health Gap Action Programme
MHSSS: Mental Health Service Satisfaction Scale
PHC: primary healthcare
SMD: severe mental disorder
WHO: World Health Organisation

## References

[1] Wang PS, Angermeyer M, Borges G, Bruffaerts R, Chiu WT, de Girolamo G (2007). Delay and failure in treatment seeking after first onset of mental health disorders in the World Health Organization’s World Mental Health Survey Initiative. World Psychiatry.

[2] Alem A, Kebede D, Fekadu A, Shibre T, Fekadu D, Beyero T (2009). Clinical course and outcome of schizophrenia in a predominantly treatment-naive cohort in rural Ethiopia. Schizophr Bull.

[3] Whiteford HA, Degenhardt L, Rehm J, Baxter AJ, Ferrari AJ, Erskine HE (2013). Global burden of disease attributable to mental and substance use disorders: findings from the Global Burden of Disease Study 2010. Lancet.

[4] Abdulahi H, Haile-Mariam D, Kebede D (2001). Burden of disease analysis in rural Ethiopia. Ethiop Med J.

[5] Kebede D, Alem A, Shibre T, Negash A, Fekadu A, Fekadu D (2003). Onset and clinical course of schizophrenia in Butajira-Ethiopia. A community-based study. Soc Psychiatry Psychiatr Epidemiol.

[6] Kebede D, Alem A, Shibire T, Deyassa N, Negash A, Beyero T (2006). Symptomatic and functional outcome of bipolar disorder in Butajira. Ethiopia J Affect Disord.

[7] Kebede D, Alem A, Shibre T, Negash A, Deyassa N, Beyero T (2005). Short-term symptomatic and functional outcomes of schizophrenia in Butajira. Ethiopia Schizophr Res.

[8] Shibre T, Kebede D, Alem A, Negash A, Deyassa N, Fekadu A (2003). Schizophrenia: illness impact on family members in a traditional society - rural Ethiopia. Soc Psychiatry Psychiatr Epidemiol.

[9] Shibre T, Negash A, Kullgren G, Kebede D, Alem A, Fekadu A (2001). Perception of stigma among family members of individuals with schizophrenia and major affective disorders in rural Ethiopia. Soc Psychiatry Psychiatr Epidemiol.

[10] Drew N, Funk M, Tang S, Lamichhane J, Chávez E, Katontoka S (2011). Human rights violations of people with mental and psychosocial disabilities: an unresolved global crisis. Lancet.

[11] Zergaw A, Hailemariam D, Alem A, Kebede D (2008). A longitudinal comparative analysis of economic and family caregiver burden due to bipolar disorder. Afr J Psychiat.

[12] Lijalem M, Tesfaye F, Alem A, Kumie A, Kebede D (2003). Nutrition status of cases of schizophrenia and bipolar disorders in Butajira, rural Ethiopia.

[13] Fekadu A, Medhin G, Kebede D, Alem A, Cleare A, Prince M (2015). Excess mortality in severe mental disorders: a 10-year population-based cohort study in rural Ethiopia. Br J Psychiatry.

[14] Mogga S, Prince M, Alem A, Kebede D, Stewart R, Glozier N (2006). Outcome of major depression in Ethiopia: population-based study. Br J Psychiatry.

[15] mhGAP-Ethiopia Working Group (2010). Mental Health Gap Action Programme in Ethiopia: final document.

[16] Federal Democratic Republic of Ethiopia Ministry of Health (2012). National Mental Health Strategy, 2012/13-2015/16.

[17] World Health Organization (2011). Mental Health Atlas: Ethiopia.

[18] Central Statistical Authority (CSA) (2008). Summary and statistical report of the 2007 population and housing census. Population size by age and sex.

[19] World Health Organization (2008). Task shifting: Global recommendations and guidelines.

[20] Fulton B, Scheffler R, Sparkes S, Auh E, Vujicic M, Soucat A (2011). Health workforce skill mix and task shifting in low income countries: a review of recent evidence. Hum Resour Health.

[21] World Health Organization (2008). mhGAP Mental Health Gap Action Programme: scaling up care for mental, neurological and substance use disorders.

[22] World Health Organization (2010). mhGAP intervention guide for mental, neurological and substance use disorders in non-specialized health settings: mental health Gap Action Programme (mhGAP).

[23] World Health Organization and Wonca (2008). Integrating mental health into primary care. A global perspective.

[24] Padmanathan P, De Silva M (2013). The acceptability and feasibility of task-sharing for mental healthcare in low and middle income countries: A systematic review. Soc Sci Med.

[25] Cohen A (2001). The effectiveness of mental health services in primary care: the view from the developing world.

[26] Hanlon C, Wondimagegn D, Alem A (2010). Lessons learned in developing community mental health care in Africa. World Psychiatry.

[27] Purgato M, Adams C, Barbui C (2012). Schizophrenia trials conducted in African countries: a drop of evidence in the ocean of morbidity?. Int J Ment Health Systems.

[28] Collins PY, Patel V, Joestl SS, March D, Insel TR, Daar AS (2011). Grand challenges in global mental health. Nature.

[29] Lund C, Alem A, Schneider M, Hanlon C, Ahrens J, Bandawe C (2015). Generating evidence to narrow the treatment gap for mental disorders in sub-Saharan Africa: rationale, overview and methods of AFFIRM. Epidemiol Psychiatr Sci.

[30] Schneider M, Baron E, Davies T, Bass J, Lund C (2015). Making assessment locally relevant: measuring functioning for maternal depression in Khayelitsha. Cape Town Soc Psychiatry Psychiatr Epidemiol.

[31] Lund C, Schneider M, Davies T, Nyatsanza M, Honikman S, Bhana A (2014). Task sharing of a psychological intervention for maternal depression in Khayelitsha. South Africa: study protocol for a randomized controlled trial Trials.

[32] Overall J, Gorham D (1962). The Brief Psychiatric Rating Scale. Psychol Rep..

[33] Robins LN, Wing J, Wittchen HU, Helzer JE, Babor TF, Burke J (1988). The Composite International Diagnostic Interview. An epidemiologic instrument suitable for use in conjunction with different diagnostic systems and in different cultures. Arch Gen Psychiatry.

[34] Shibre T, Kebede D, Alem A, Negash A, Kibreab S, Fekadu A (2002). An evaluation of two screening methods to identify cases with schizophrenia and affective disorders in a community survey in rural Ethiopia. Int J Soc Psychiatry.

[35] WHO (1997). Schedules for Clinical Assessment in Neuropsychiatry, version 2.1.

[36] Keller MB, Lavori PW, Friedman B (1987). The longitudinal interval follow-up evaluation. A comprehensive method for assessing outcome in prospective longitudinal studies. Arch Gen Psychiatry.

[37] Araya M, Mussie M, Jacobsson L (2009). Decentralized psychiatric nursing service in Ethiopia--a model for low income countries. Ethiop Med J.

[38] American Psychiatric Association (1994). Diagnostic and Statistical Manual of Mental Disorders (4th Edition) (DSM-IV).

[39] Andrews G, Henderson S, Hall W. Overview of the Australian National Mental Health Survey. Prevalence, comorbidity, disability and service utilisation. Br J Psychiatr. 2001;178:145-153 10.1192/bjp.178.2.145 11157427

[40] Mayston R, Alem A, Habtamu A, Shibre T, Fekadu A, Hanlon C. Participatory planning of a primary care service for people with severe mental disorders in rural Ethiopia. Health Policy Planning. 2015:doi:10.1093/heapol/czv072. 10.1093/heapol/czv072 PMC5007595 26282860

[41] Shibre T, Medhin G, Alem A, Kebede D, Teferra S, Jacobsson L (2014). Long-term clinical course and outcome of schizophrenia in rural Ethiopia: 10-year follow-up of a population-based cohort. Schizophr Res.

[42] McGuffin P, Farmer A, Harvey I (1991). A polydiagnostic application of operational criteria in studies of psychotic illness: Development and reliability of the opcrit system. Arch Gen Psychiatry.

[43] Hanlon C, Luitel NP, Kathree T, Murhar V, Shrivasta S, Medhin G (2014). Challenges and Opportunities for Implementing Integrated Mental Health Care: A District Level Situation Analysis from Five Low- and Middle-Income Countries. PLoS One.

[44] Mall S, Hailemariam M, Selamu M, Fekadu A, Lund C, Patel V et al. “Restoring the person’s life”: a qualitative study to inform development of care for people with severe mental disorders in rural Ethiopia. Epidemiology and Psychiatric Sciences. 2015: In press. 10.1017/S2045796015001006 PMC6998647 26961343

[45] Gureje O, Lasebikan VO, Ephraim-Oluwanuga O, Olley BO, Kola L (2005). Community study of knowledge of and attitude to mental illness in Nigeria. Br J Psychiatry.

[46] Kohrt BA, Jordans MJD, Rai S, Shrestha P, Luitel NP, Ramaiya MK (2015). Therapist competence in global mental health: Development of the ENhancing Assessment of Common Therapeutic factors (ENACT) rating scale. Behav Res Ther.

[47] Lukoff D, Nuechterlein KH, Ventura J (1986). Symptom monitoring in the rehabilitation of schizophrenic patients. Schizophr Bull.

[48] Burlingame GM, Seaman S, Johnson JE, Whipple J, Richardson E, Rees F (2006). Sensitivity to change of the Brief Psychiatric Rating Scale - Extended (BPRS-E): an item and subscale analysis. Psychol Serv.

[49] Wykes T, Parr A-M, Landau S (1999). Group treatment of auditory hallucinations. Br J Psychiatry.

[50] Van der Does AJW, Dingemans PMAJ, Linszen DH, Nugter MA, Scholte WF (1996). Symptoms, cognitive and social functioning in recent-onset schizophrenia: a longitudinal study. Schizophr Res.

[51] Youngmann R, Zilber N, Workneh F, Giel R (2008). Adapting the SRQ for Ethiopian populations: a culturally sensitive psychiatric screening instrument. Transcult Psychiatry.

[52] Burlingame GM, Dunn TW, Chen S, Lehman A, Axman R, Earnshaw D (2005). Selection of outcome assessment instruments for inpatients with severe and persistent mental illness. Psychiatr Serv.

[53] Schumi J, Wittes JT (2011). Through the looking glass: understanding non-inferiority. Trials.

[54] Jacobson NS, Truax P (1991). Clinical significance: a statistical approach to defining meaningful change in psychotherapy research. J Consult Clin Psychol.

[55] Gonda T, Deane FP, Murugesan G. Predicting clinically significant change in an inpatient program for people with severe mental illness Australian and New Zealand Journal of Psychiatry. 2012:doi: 10.1177/0004867412445527 10.1177/0004867412445527 22528976

[56] Rosenheck R, Lin H (2014). Noninferiority of perphenazine vs. three second-generation antipsychotics in chronic schizophrenia. J Nerv Ment Dis.

[57] Gray R, Leese M, Bindman J, Becker T, Burti L, David A (2006). Adherence therapy for people with schizophrenia. European multicentre randomised controlled trial. Br J Psychiatry.

[58] Pocock SJ (2006). Clinical trials: a practical approach.

[59] Adams G, Gulliford MC, Ukoumunne OC, Eldridge S, Chinn S, Campbell MJ (2004). Patterns of intra-cluster correlation from primary care research to inform study design and analysis. J Clin Epidemiol.

[60] Piaggio G, Elbourne DR, Altman DG, Pocock SJ (2006). Evans SJW, for the CONSORT group. Reporting of noninferiority and equivalence randomized trials: an extension of the CONSORT statement JAMA.

[61] World Health Organization (WHO). World Health Organisation Disability Assessment Schedule II. 12 item interviewer-administered version. Geneva: 2000.

[62] Susser E, Finnerty M, Mojtabai R, Yale S, Conover S, Goetz R (2000). Reliability of the Life Chart Schedule for assessment of the long-term course of schizophrenia. Schizophr Res.

[63] Chisholm D, Sekar K, Kishore K, Saeed K, James S, Mubbashar M (2000). Integration of mental health care into primary care: demonstration of cost-outcome study in India and Pakistan. Br J Psychiatry.

[64] Beecham J, Knapp M (2001). Costing psychiatric interventions. Measuring Mental Health Needs.

[65] Weiden P, Miller A (2001). Which side effects really matter? Screening for common and distressing side-effects of antipsychotic medications. J Psychiatr Pract.

[66] Morisky DE, Ang A, Krousel-Wood M, Ward HJ (2008). Predictive validity of a medication adherence measure in an out-patient setting. J Clin Hypertens.

[67] Lysaker PH, Roe D, Yanos PT (2007). Toward Understanding the Insight Paradox: Internalized Stigma Moderates the Association Between Insight and Social Functioning, Hope, and Self-esteem Among People with Schizophrenia Spectrum Disorders. Schizophr Bull.

[68] Priebe S, Grutyers T (1993). The role of the helping alliance in psychiatric community care: a prospective study. Journal of Nervous & Mental Disease.

[69] Sartorius N, Janca A (1996). Psychiatric assessment instruments developed by the World Health Organization. Social Psychiatry & Psychiatric Epidemiology.

[70] Hodgson R, Alwyn T, John B, Thom B, Smith A (2002). The FAST Alcohol Screening Test. Alcohol Alcohol.

