# Correction to: Task sharing for the care of severe mental disorders in a low-income country (TaSCS): study protocol for a randomised, controlled, non-inferiority trial

**DOI:** 10.1186/s13063-020-04776-3

**Published:** 2020-10-06

**Authors:** Charlotte Hanlon, Atalay Alem, Girmay Medhin, Teshome Shibre, Dawit A. Ejigu, Hanna Negussie, Michael Dewey, Lawrence Wissow, Martin Prince, Ezra Susser, Crick Lund, Abebaw Fekadu

**Affiliations:** 1 Addis Ababa University, College of Health Sciences, School of Medicine, Department of Psychiatry, Addis Ababa, Ethiopia; 2 Institute of Psychiatry, Psychology and Neuroscience, Centre for Global Mental Health, King’s College, London, UK; 3 Aklilu Lemma Institute of Pathobiology, Addis Ababa University, Addis Ababa, Ethiopia; 4 Horizon Health Network, Dr Everett Chalmers Regional Hospital, Psychiatry, Fredericton, New Brunswick Canada; 5 Department of Pharmacology, St Paul’s Hospital Millennium Medical College, Addis Ababa, Ethiopia; 6 Department of Health, Behaviour and Society, Johns Hopkins School of Public Health, Baltimore, MD USA; 7 Mailman School of Public Health, Columbia University, New York, USA; 8 New York State Psychiatric Institute, New York, USA; 9 Department of Psychiatry and Mental Health, Alan J. Flisher Centre for Public Mental Health, University of Cape Town, 46 Sawkins Road, Rondebosch, Cape Town, South Africa; 10 Department of Psychological Medicine, Institute of Psychiatry, King’s College London, Psychology and Neuroscience, Centre for Affective Disorders, London, UK

**Correction to: Trials 17, 76 (2016)**

**https://doi.org/10.1186/s13063-016-1191-x**

Following publication of the original article [1], the authors notified us that they mistakenly indicated they would report medication adherence as a trial outcome measured by the Morisky Medication Adherence Scale. However, the authors will no longer use this measure of adherence in the TaSCS trial analysis.

## References

[1] Hanlon C (2020). Task sharing for the care of severe mental disorders in a low-income country (TaSCS): study protocol for a randomised, controlled, non-inferiority trial. Trials.

