# Supplementary material for: Task sharing for the care of severe mental disorders in a low-income country (TaSCS): study protocol for a randomised, controlled, non-inferiority trial
Source: Trials. 2016 Feb 11;17:76. doi: 10.1186/s13063-016-1191-x (PMC4750210; doi:10.1186/s13063-016-1191-x)
Supplement: Additional file 1: — Operationalisation and assessment of eligibility criteria. Description: Specifies the eligibility criteria, together with the measures for assessing eligibility. (DOCX 14 kb) [file 13063_2016_1191_MOESM1_ESM.docx]

Additional File 1: Operationalisation and assessment of eligibility criteria

| **Inclusion criteria** | **Operationalisation** |
| --- | --- |
| Capacity to consent or guardian permission in absence of active refusal | Structured psychiatric nurse assessment |
| DSM-IV[^34^](#_ENREF_34) diagnosis of schizophrenia, schizoaffective disorder, bipolar disorder, major depressive disorder | Schedules of Clinical Assessment in Neuropsychiatry for Butajira SMD cohort[^31^](#_ENREF_31).  Standardised semi-structured assessment for Butajira hospital psychiatric clinic sample[^31^](#_ENREF_31). |
| Need for continuing mental healthcare: |  |
| Prescription of psychotropic medication within the past 2 years | Life Chart Schedule[^55^](#_ENREF_55) |
| Suicide attempt within last 2 years |  |
| Violence against others in last 2 years |  |
| Relapse or not in complete remission in last 2 years |  |
| Admitted as an in-patient for psychiatric care in last 2 years |  |
| **Exclusion criteria (Phases 1 and 2)** |  |
| Current suicidal intent | Mini International Neuropsychiatric Interview score of 17 or more or endorses any item indicating suicidal intent (B6, 8, 9, 10 or 12)[^56^](#_ENREF_56) |
| **Exclusion criteria (Phase 1 only)** |  |
| Symptomatic at the time of assessment | Brief Psychiatric Rating Scale-Expanded version score of 52 or more[^57^](#_ENREF_57) |
| Evidence of unstable illness in the past 3 months |  |
| Suicide attempt in last 3 months | Life chart schedule[^55^](#_ENREF_55) |
| Violence against another person in last 3 months |  |
| Victim of violence from another person in last 3 months |  |
| Alcohol use disorder within last 12 months | Fast Alcohol Screening Test[^58^](#_ENREF_58) |
| Khat use disorders within last 12 months | Composite International Diagnostic Interview[^29^](#_ENREF_29) |
| Co-morbid medical condition | Structured history and physical assessment by psychiatric nurse |
