# Supplementary material for: Task sharing for the care of severe mental disorders in a low-income country (TaSCS): study protocol for a randomised, controlled, non-inferiority trial
Source: Trials. 2016 Feb 11;17:76. doi: 10.1186/s13063-016-1191-x (PMC4750210; doi:10.1186/s13063-016-1191-x)
Supplement: Additional file 2: — Roles of primary care workers in the task sharing intervention service. Description: Describes the roles that the primary care workers are being trained to do within the new task-shared service. (DOCX 12 kb) [file 13063_2016_1191_MOESM2_ESM.docx]

Additional File 2: Roles of primary care workers in the task sharing intervention service

| **Health Centre nurses and health officers** | **Health extension workers** |
| --- | --- |
| **Role**   - Establish rapport and therapeutic alliance - Monitor response to treatment - Adjust medication as needed - Manage side effects of medication - Detect and manage non-adherence - Monitor suicide risk - Psychoeducate patient and caregivers - Address current psychosocial stressors - Reactivate social networks - Provide support to patient and caregivers - Conduct a physical health review - Screen and brief intervention for co-morbid substance misuse - Provide regular follow-up (minimum 3-monthly) - Detect those who drop out of care and arrange outreach by HEW - Know when to refer for specialist review | **Role**   - Establish therapeutic rapport - Psychoeducate and support patient and caregivers - Facilitate rehabilitation - Support adherence and engagement with care - Detect relapse - Detect side effects of medication - Provide outreach to those who drop out of care - Awareness-raising within community to counter stigma, discrimination and abuse - Know when to refer to health centre |
| **Referral / consultation criteria**   - Active suicide intent - Inadequate food/fluid intake - Persistently non-adherent to medication - Acutely disturbed and not manageable at home - Pregnancy/breastfeeding - Medical complications - Unmanageable side effects - 2 or more relapses in 12 months prior to review (indicating poor control) - Treatment-resistance | **Referral criteria**   - Active suicide intent - Inadequate food / fluid intake - Persistently non-adherent to medication - Acutely disturbed and not manageable at home - Pregnancy / breastfeeding - Medical complications - Unmanageable side effects |
