# Supplementary material for: Task sharing for the care of severe mental disorders in a low-income country (TaSCS): study protocol for a randomised, controlled, non-inferiority trial
Source: Trials. 2016 Feb 11;17:76. doi: 10.1186/s13063-016-1191-x (PMC4750210; doi:10.1186/s13063-016-1191-x)
Supplement: Additional file 3: — Planned data analyses for secondary outcomes. Description: Gives details of the analysis plan for secondary trial outcomes. (DOCX 12 kb) [file 13063_2016_1191_MOESM3_ESM.docx]

Additional File 3: Planned data analyses for secondary outcomes

| ***Secondary outcome*** | ***Measure*** | | ***Type of data*** | | ***Proposed model for data analysis*** |
| --- | --- | --- | --- | --- | --- |
| Functioning | Adapted WHODAS 2.0 and local functioning scale | | Count | | Zero-inflated negative binomial regression |
| Relapse | LCS | | Count / binary | | Poisson / logistic regression |
| Service use costs | CSRI | | Positively skewed, continuous | | Linear regression using bootstrap methods |
| Service satisfaction | MHSSS | | Continuous | | Linear regression |
| Underweight | Body Mass Index < 17.5kg/m^2^ | | Binary | | Logistic regression |
| Service use for physical health care | CSRI – any investigation or referral for physical health problem | | Binary | | Logistic regression |
| Medication side effects | ASC: any side effect | | Binary | | Logistic regression |
| Adherence to medication | Medication Adherence Measure: any non-adherence | | Binary | | Logistic regression |
| Patient engagement | Dropped out of care or missed more than 50% of appointments | | Binary | | Logistic regression |
| Perceived stigma | Score above median on the Internalised Stigma in Mental Illness scale | | Binary | | Logistic regression |
| Restraint | Proportion chained or restrained | | Binary | | Logistic regression |
| Quality of care | Number of non-evidence-based clinical interventions | | Count | | Poisson regression |
| Serious adverse events | Any hospital admission for medical emergency | | Binary | | Logistic regression |
|  | Any deaths | | Binary | | Logistic regression |
| **Caregivers** |  | |  | |  |
| Perceived stigma | Score above median on the Family Interview Schedule stigma items | Binary | | Logistic regression | |
| Caregiver burden | Score above median on the Family Interview Schedule carer burden | Binary | | Logistic regression | |
| Time and opportunity costs | CSRI | Positively skewed, continuous | | Linear regression using bootstrap methods | |
